# Supplementary material for: Molecular basis and design principles of switchable front-rear polarity and directional migration in Myxococcus xanthus
Source: Nat Commun. 2023 Jul 8;14:4056. doi: 10.1038/s41467-023-39773-y (PMC10329633; doi:10.1038/s41467-023-39773-y)
Supplement: Supplementary file 1 — Supplementry Information [file 41467_2023_39773_MOESM1_ESM.pdf]

Supplementary Information

Molecular basis and design principles of switchable front-rear polarity and directional migration in *Myxococcus xanthus*

Luís António Menezes Carreira, Dobromir Szadkowski, Stefano Lometto, Georg A.K. Hochberg & Lotte Søgaard-Andersen

This file includes

- Supplementary Figure 1-8
- Supplementary Tables 1-4
- Supplementary References

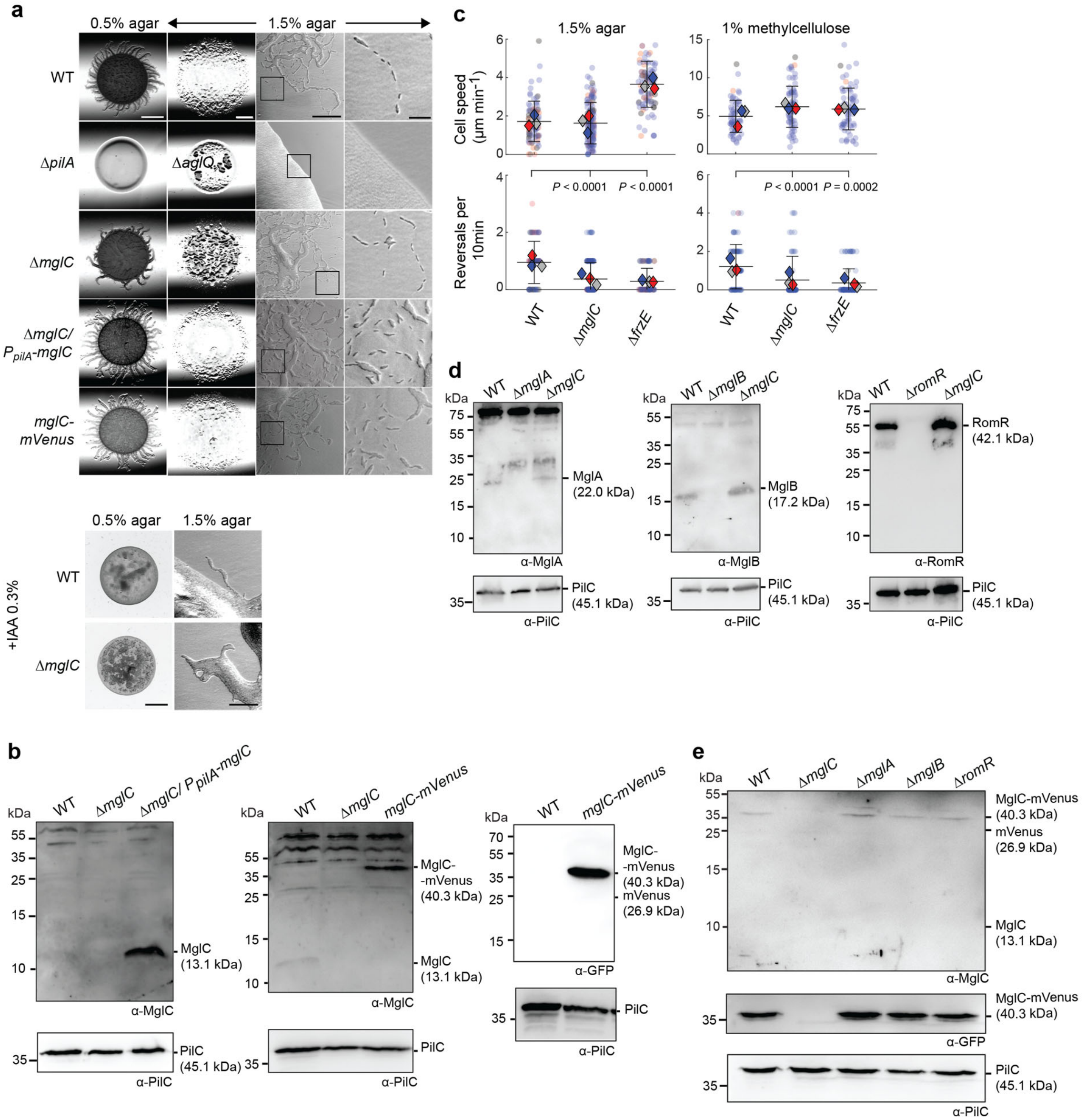

<u>Supplementary Fig. 1. MglC is important for the correct reversal frequency</u>

a. MglC is important for T4P-dependent motility and gliding. Cells were incubated on 0.5% agar/0.5% CTT and 1.5% agar/0.5% CTT to score T4P-dependent and gliding motility, respectively and in the absence or presence of IAA. Scale bars, 1mm (0.5% agar), 1mm (1.5% agar, left), 100µm (1.5% agar, center) and 20µm (1.5% agar, right). In the Δ*mglC*/P*pilA-mglC* complementation strain, *mglC* was expressed from the *pilA* promoter on a plasmid integrated in a single copy at the Mx8 *attB* site*. mglC-mVenus* is integrated at the native site. T4P-dependent motility and gliding motility is favored on 0.5% and 1.5% agar, respectively[1]. T4P-dependent motility is characterized by the formation of flares at the edge of colonies and gliding by the presence of single cells at the colony edge. The squares in the third column figures indicate the original location from which the figures in the fourth column were generated. Similar results were obtained in three independent experiments. **b.** Immunoblot analysis of MglC and MglC-mVenus accumulation. Lysates from the same number of cells were separated by SDS-PAGE and probed with α-MglC or α-GFP antibodies and after stripping with α-PilC antibodies (loading control). Calculated molecular masses of the indicated proteins are indicated on the right and molecular weight masses of markers on the left. Similar results were obtained in two independent experiments. **c.** MglC is important

for the correct reversal frequency. Speed (upper panels) and reversals (lower panels) of single cells moving by gliding motility (1.5% agar, left) or T4P-dependent motility (covered with 1% methylcellulose, right) were determined. Data are shown for three biological replicates with the following number of cells analyzed per strain: Cell speed and reversal frequency on 1.5% agar (WT: 23, 26, 29; Δ*mglC*: 38, 32, 25; Δ*frzE*: 54, 47, 43); and 1% methylcellulose (WT: 21, 24, 20; Δ*mglC*: 25, 27, 22; Δ*frzE*: 21, 23, 29). Individual data points are shown in red, blue and grey and the corresponding mean (diamond) in the same color. Error bars indicate the mean ± standard deviation (STDEV). The precise *P* values in two-sided Student's *t*-test are included in the Source Data file. **d, e.** Immunoblot analysis of MglA, MglB, RomR and MglC-mVenus accumulation.
Immunoblot analysis was done as in Fig. S1b. Similar results were obtained in two independent experiments.

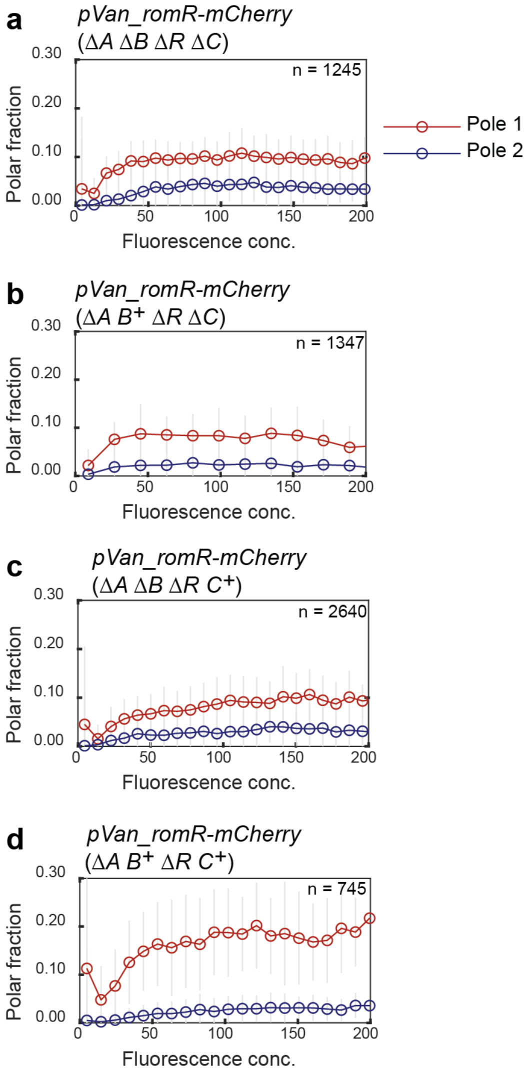

<u>Supplementary Fig. 2. MglC is not important for cooperative RomR polar accumulation</u>

a-d. Induction of *romR-mCherry* expression from the vanillate inducible promoter in Δ*mglA* Δ*mglB* Δ*romR* Δ*mglC* cells (a), Δ*mglA* Δ*romR* Δ*mglC* cells (b), Δ*mglA* Δ*mglB* Δ*romR* cells (c) and Δ*mglA* Δ*romR* cells (d). All strains contain the Δ*aglQ* Δ*frzE* mutations to allow monitoring of the same cells for 6h and to reduce Frz-dependent polarity inversions. n, number of individual cells observed. In the diagrams, the poles with the highest and lowest polar fraction of fluorescence are defined as pole 1 and pole 2, respectively. Cells were grown in the absence of vanillate and then placed on an agar surface containing 300μM vanillate and imaged for 6h. Cells from all frames of the time-lapse recordings were pooled and binned according to their fluorescence concentration (total cellular fluorescence divided by cell area). Plotted are the mean ± 1 STDEV of all cells within each bin. Each bin contain data from at least five cells. Data in c and d are from[2]. The shape of the polar accumulation curves provides evidence for positive cooperativity in RomR-mCherry polar localization[2]: In the absence of any cooperativity, individual RomR-mCherry molecules would localize independently of one another, such that the fraction at each pole should be constant and independent of concentration. Instead, the fractions of RomR-mCherry at both poles increase with fluorescence concentration, supporting that RomR-mCherry self-recruits or stabilizes its polar accumulation.

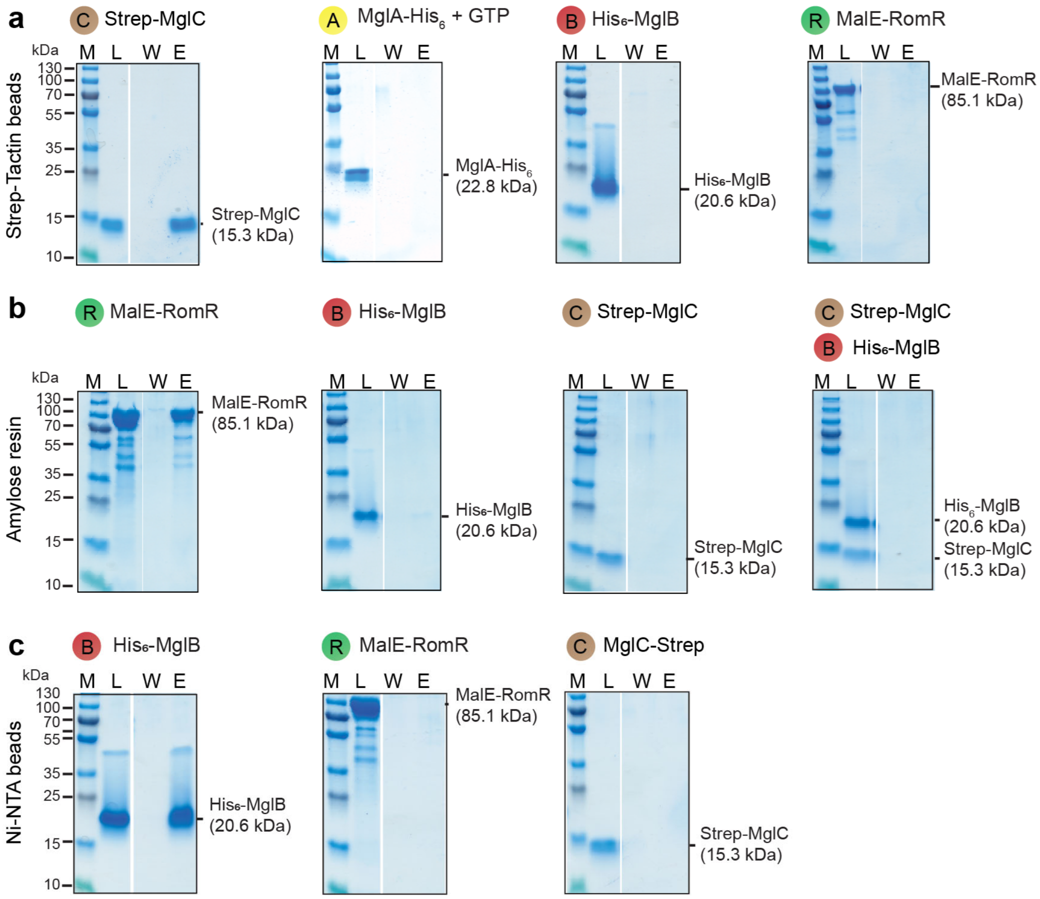

<u>Supplementary Fig. 3. *In vitro* pull-down experiments with MglA-His$_6$, His$_6$-MglB, Strep-MglC and MalE-RomR</u>

a-c. Control experiments to the experiments shown in Fig. 2. Proteins were mixed as indicated at final concentrations of 10µM and applied to the indicated matrices. Matrices were washed and bound proteins eluted. In experiments with MglA-His$_6$, the protein was preloaded with GTP and all buffers contained 40µM GTP. Equivalent volumes of the load (L), last wash (W) and elution fraction (E) were separated on the same SDS-PAGE gel and stained with Coomassie Brilliant Blue. Gap between lanes indicates lanes deleted for presentation purposes. Calculated molecular masses of the indicated proteins are indicated on the right. Molecular weight markers (M) are indicated on the left. For samples with MalE-RomR, the faster migrating proteins are degradation products. Similar results were obtained in three independent experiments.

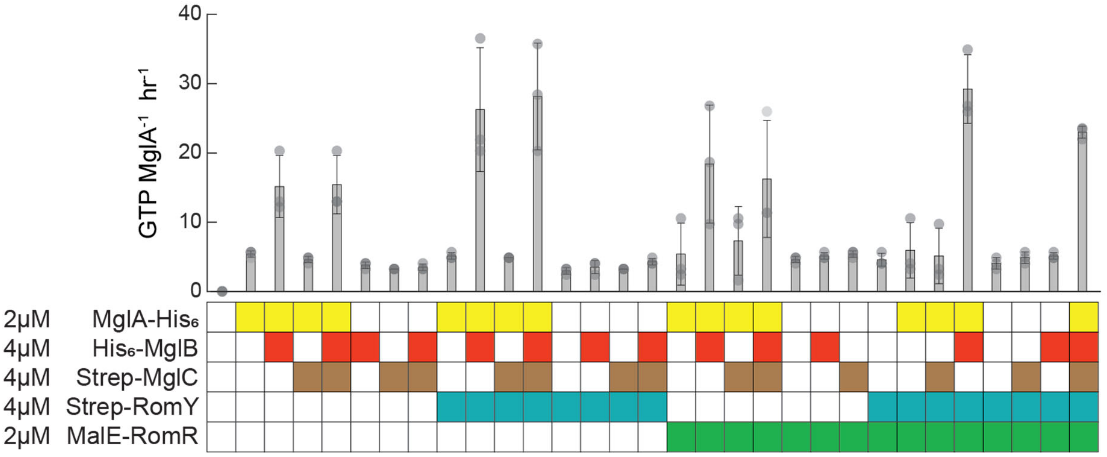

<u>Supplementary Fig. 4. RomR, MglC and RomR/MglC do not have MglA GAP activity and do not affect MglB and MglB/RomY GAP activity.</u>
The indicated proteins at the indicated concentrations were mixed and GTPase activity measured in a continuous, regenerative coupled GTPase assay after 1h of incubation. Boxes below diagrams indicate the presence (colored box) or absence (white box) of a protein. MglA-His_6 was preloaded with GTP (final concentration 3.3mM). Data are shown for three technical replicates (grey dots) and as mean ± STDEV from the three technical replicates.

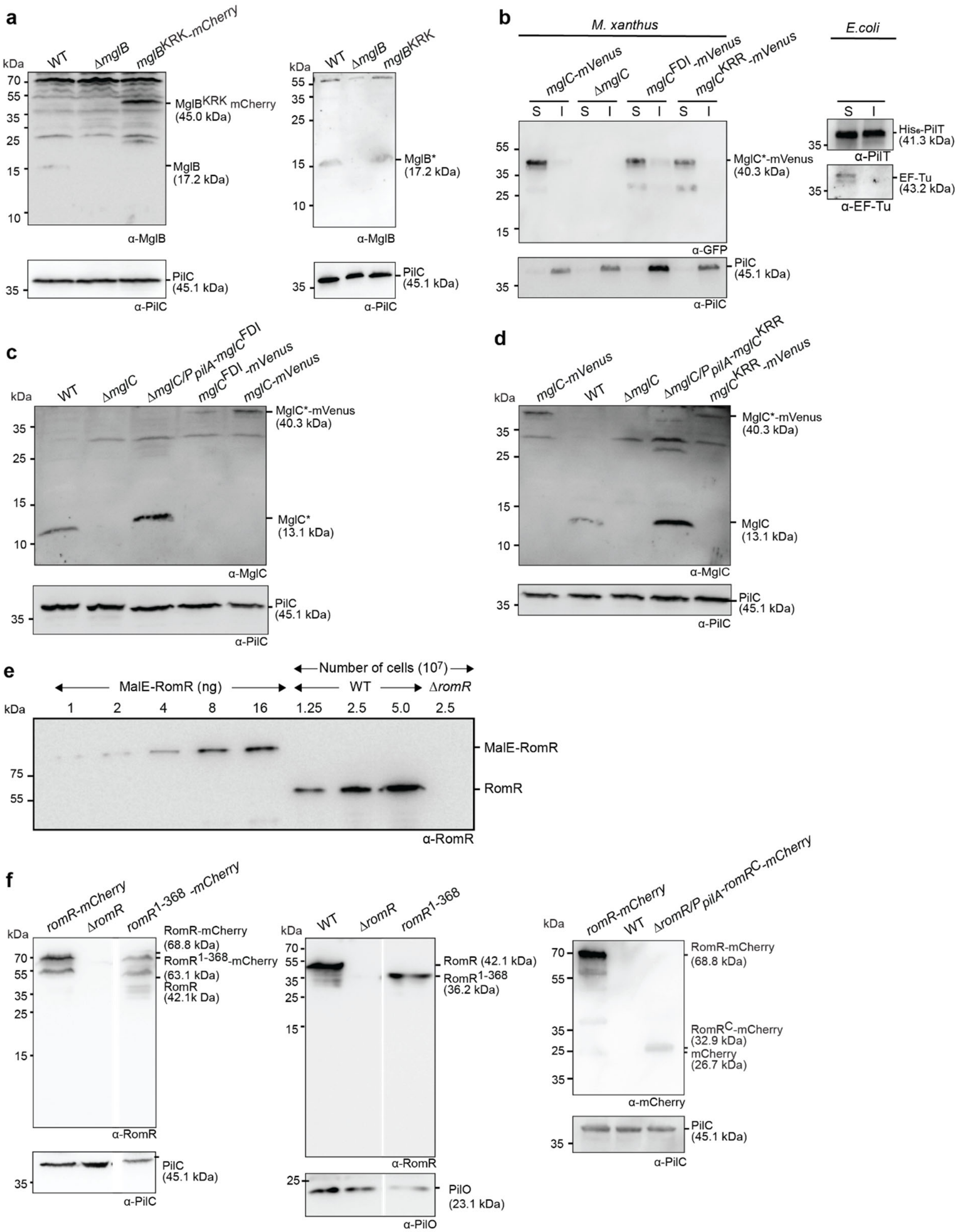

<u>Supplementary Fig. 5. Analysis of the accumulation of MglB, MglC and RomR variants</u>

a, c, d, f. Immunoblot analysis of accumulation of MglB and MglB-mCherry variants (a), MglC and MglC-mVenus variants (c-d), and RomR variants (f). PilC or PilO were used as loading controls. Immunoblot analysis was done as in Fig. S1b. All mutant alleles were expressed from the relevant native site except for those labelled P*pilA*, which were expressed from the *pilA* promoter on a plasmid integrated in a single copy at the Mx8 *attB* site. Similar results were obtained in two independent experiments.

b. MglC-mVenus, $MglC^{FDI}$-mVenus and $MglC^{KRR}$-mVenus are soluble proteins. Cell lysates of *M. xanthus* strains expressing the indicated fusion proteins were fractionated into fractions enriched for soluble and insoluble proteins including inner membrane and outer membrane proteins (Methods). Proteins in the soluble (S) and insoluble (I) fractions were separated by SDS-PAGE and analyzed by immunoblotting with α-GFP antibodies and after stripping with α-PilC antibodies. Calculated molecular masses of the indicated proteins are indicated on the right and molecular weight masses of markers on the left. PilC is an inner membrane protein[3] and was used as a control for the insoluble fraction. As a control for a protein that forms inclusion bodies, His_6-PilT[4] expressed in *E. coli* was used. EF-Tu was used a control for a soluble protein in *E.coli*. Similar results were obtained in two independent experiments.
e. Quantification of RomR molecules per cell by immunoblotting. Cell extracts from the indicated number of WT *M. xanthus* cells or Δ*romR* cells were probed with specific α-RomR antibodies in parallel with different amounts of purified MalE-RomR. Molecule number per cell was calculated from the intensity of the band in WT lysates compared to a standard curve prepared from the dilution series of known amounts of MalE-RomR on the same blot. Concentrations were calculated based on three independent immunoblots. Shown is a representative blot.

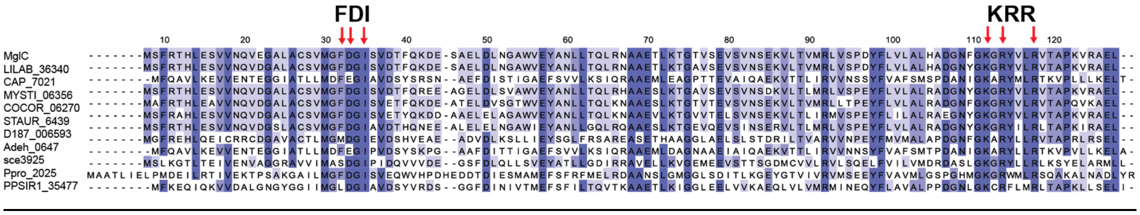

Supplementary Fig. 6. Alignment of MglC homologs
Identity is indicated in shades of blue. The FDI and KRR residues are indicated with red arrows.

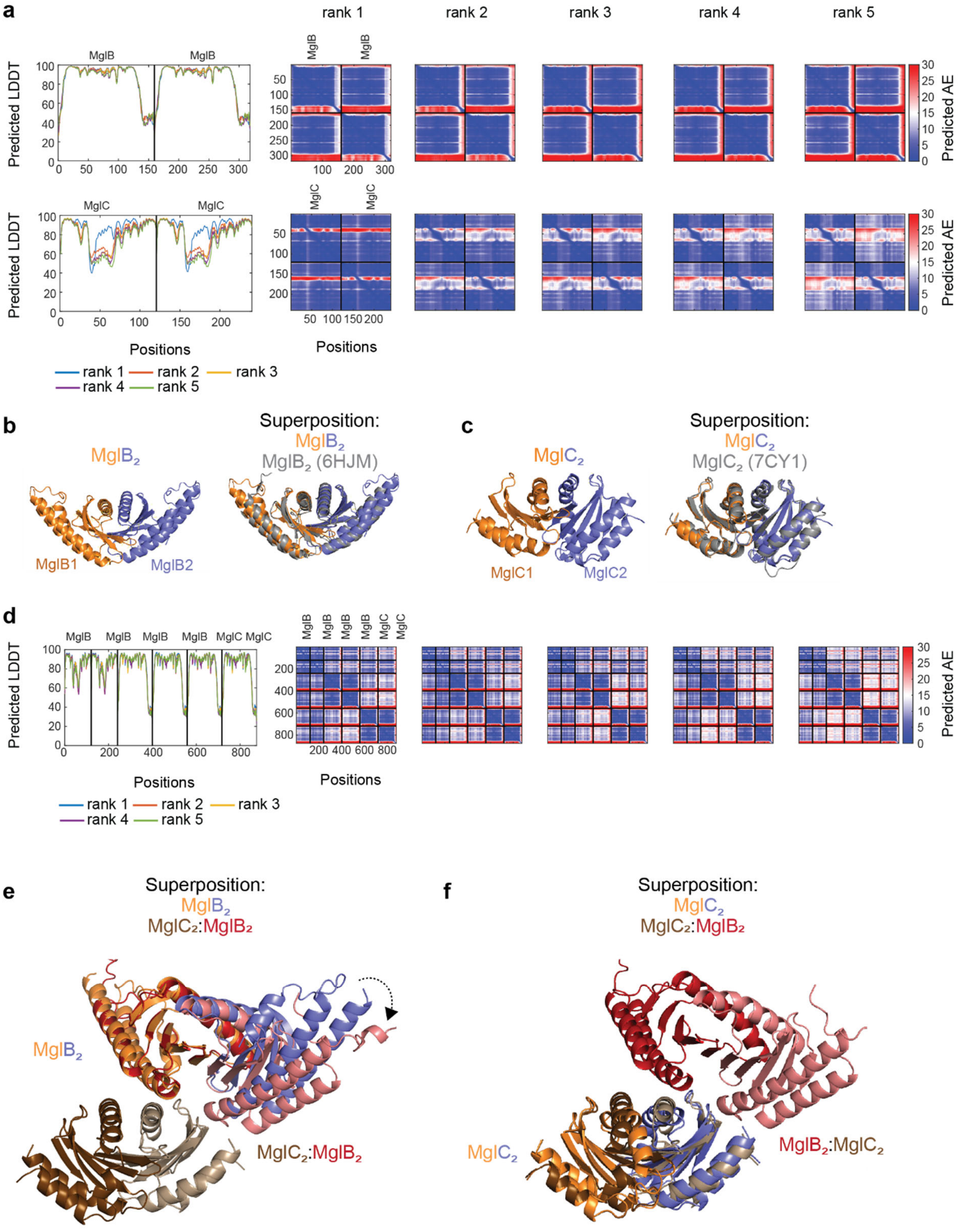

Supplementary Fig. 7. AlphaFold-Multimer models of dimeric MglB, dimeric MglC and the $(MglB_2)_2$:$MglC_2$ complex.

a. pLDDT and pAE plots of five AlphaFold-Multimer models of dimeric MglB and dimeric MglC.

b. Superposition of the rank 1 AlphaFold-Multimer model of dimeric MglB (orange and blue) on the solved structure of MglB (pdb ID: 6hjm[5]) (grey).

c. Superposition of the rank 1 AlphaFold-Multimer model of dimeric MglC (orange and blue) on the solved structure of MglC (pdb ID: 7CY1[6] (grey).
d. pLDDT and pAE plots of five AlphaFold-Multimer models of the $MglC_2$:$(MglB_2)_2$ complex. The Rank 1 model is the one used in Fig. 6a and Fig. 7c.
e. Superposition of the rank 1 AlphaFold-Multimer model of the $MglC_2$:$MglB_2$ complex (brown/red) with the AlphaFold-Multimer model of $MglB_2$ (orange and blue). The arrow indicates the repositioning of the α1/α3-helices in MglB on the four-helix side in the complex with MglC compared to MglB in isolation. This repositioning results in a more closed state of the four-helix side in the MglB dimer when complexed with $MglC_2$. For simplicity, the MglC dimer is shown to interact with only one MglB dimer.
f. Superposition of the rank 1 AlphaFold-Multimer model of the $MglC_2$:$MglB_2$ complex (brown/red) with the AlphaFold-Multimer model of $MglC_2$ (orange and blue). For simplicity, the MglC dimer is shown to interact with only one MglB dimer.

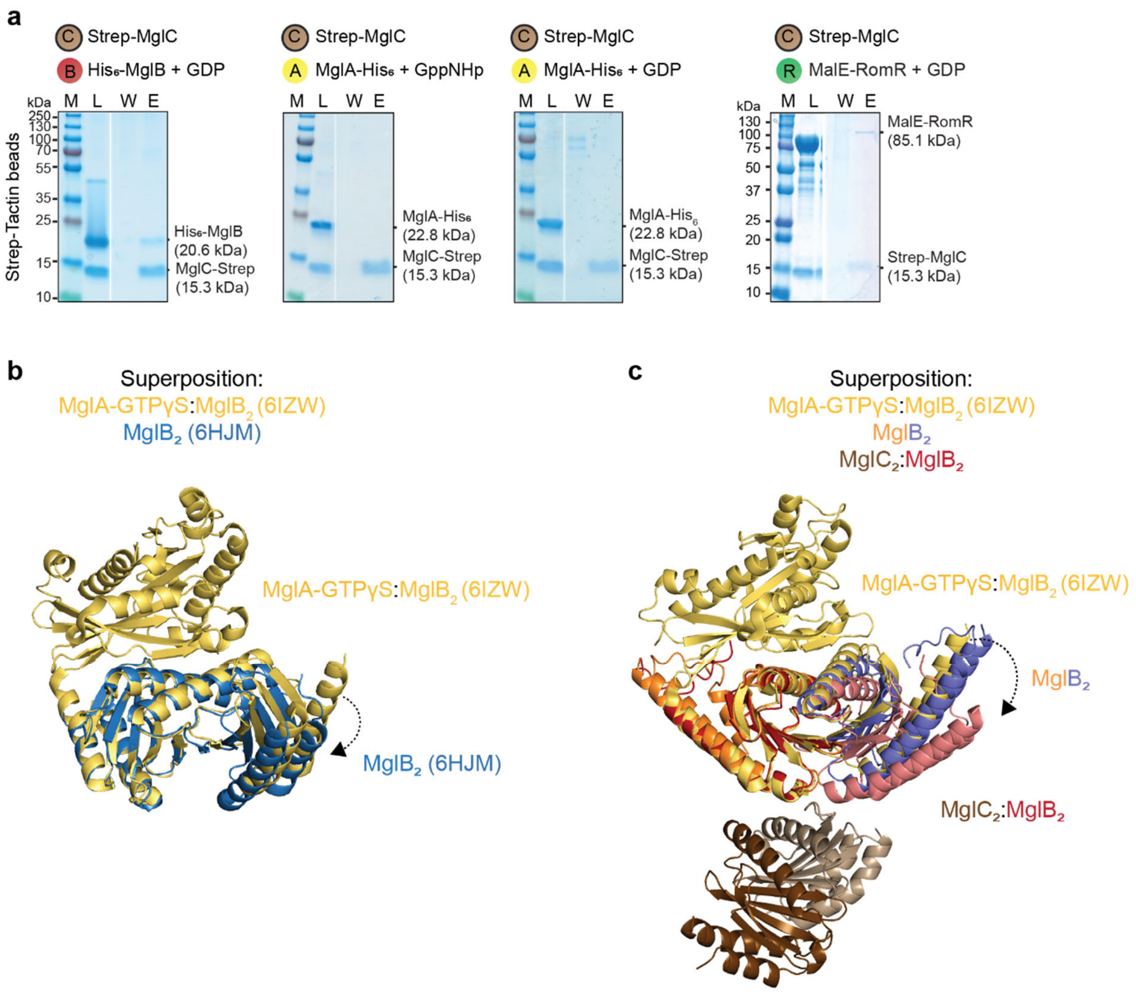

Supplementary Fig. 8. *In vitro* pull-down experiments with MglA-His$_6$, His$_6$-MglB, Strep-MglC and MalE-RomR

a. Control experiments to the experiments shown in Fig. 7a,b. Proteins were mixed as indicated at final concentrations of 10µM and applied to the indicated matrix. The matrix was washed and bound proteins eluted. The bait protein is indicated by the black circle. In experiments with MglA-His$_6$, the protein was preloaded with the indicated nucleotide and all buffers contained 40µM of the relevant nucleotide. Equivalent volumes of the load (L), last wash (W) and elution fraction (E) were separated on the same SDS-PAGE gel and stained with Coomassie Brilliant Blue. Gap between lanes indicates lanes deleted for presentation purposes. Calculated molecular masses of the indicated proteins are indicated on the right. Molecular weight markers (M) are indicated on the left. Similar results were obtained in three independent experiments.

b. Superposition of the solved structure of MglA-GTPɣS:MglB$_2$ (pdb ID: 6izw[7]) (yellow) on the solved structure of MglB$_2$ (pdb ID: 6hjm[5]) (blue). The arrow indicates the repositioning of the α1/α3-helices in MglB on the four-helix side in the MglA-GTPɣS:MglB$_2$ complex compared to MglB$_2$ in isolation. This repositioning results in a more closed state of the four-helix side in MglB$_2$ in isolation.

c. Superposition of the solved structure of MglA-GTPɣS:MglB$_2$ (pdb ID: 6izw[7]) (yellow) on the rank 1 AlphaFold-Multimer model of MglB$_2$ (orange and blue) and the rank 1 AlphaFold-Multimer model of the MglC$_2$:MglB$_2$ complex (brown/red). The arrow indicates the repositioning of the α1/α3-helices in MglB on the four-helix side from the more open

conformation in MglA-GTPγS:$MglB_2$ complex over $MglB_2$ in isolation to MglB in the $MglC_2$:$MglB_2$ complex.

Supplementary Table 1. *M. xanthus* strains used in this work

Strains	Genotype	Source or Reference
DK1622	WT	[8]
DK10410	Δ*pilA*	[9]
SA5293	Δ*aglQ*	[10]
SA7300	Δ*mglC*	[11]
SA4420	Δ*mglA*	[12]
SA3387	Δ*mglB*	[13]
SA3300	Δ*romR*	[14]
SA7301	Δ*mglA* Δ*mglC*	[11]
SA7302	Δ*mglB* Δ*mglC*	[11]
SA7304	Δ*romR* Δ*mglC*	[11]
SA8802	Δ*frzE*	[15]
SA7305	Δ*mglC* P_{pilA}-*mglC*	[11]
SA8185	*mglA-mVenus*	[15]
SA8130	*mglA-mVenus* Δ*mglC*	This work
SA6963	*mglB-mCherry*	[14]
SA8155	*mglB-mCherry* Δ*mglC*	This work
SA7507	*romR-mCherry*	[15]
SA8129	*romR-mCherry* Δ*mglC*	This work
SA10466	*mglC-mVenus*	This work
SA10627	*mglC-mVenus* Δ*mglA*	This work
SA10535	*mglC-mVenus* Δ*mglB*	This work
SA10467	*mglC-mVenus* Δ*romR*	This work
SA10627	*mglC-mVenus* Δ*mglA*	This work
SA10891	*mglC-mVenus* Δ*mglA* Δ*mglB*	This work
SA10925	*mglC-mVenus* Δ*mglA* Δ*romR*	This work
SA10954	*mglC-mVenus* Δ*mglA* Δ*mglB* Δ*romR*	This work
SA3971	*mglB-mCherry* Δ*mglA*	[14]
SA10971	*mglB-mCherry* Δ*mglA* Δ*mglC*	This work
SA10776	*mglB-mCherry* Δ*mglA* Δ*romR*	[2]
SA12836	*mglB-mCherry* Δ*mglA* Δ*mglC* Δ*romR*	This work
SA7579	*romR-mCherry* Δ*mglA*	[15]
SA10788	*romR-mCherry* Δ*mglA* Δ*mglB*	[2]
SA10898	*romR-mCherry* Δ*mglA* Δ*mglC*	This work
SA11027	*romR-mCherry* Δ*mglA* Δ*mglB* Δ*mglC*	This work
SA10807	p_{Van}-*romR-mCherry* Δ*mglA* Δ*mglB* Δ*romR* Δ*frzE* Δ*aglQ*	[2]
SA12814	p_{Van}-*romR-mCherry* Δ*mglA* Δ*mglB* Δ*mglC* Δ*romR* Δ*frzE* Δ*aglQ*	This work
SA10769	p_{Van}-*romR-mCherry* Δ*mglA* Δ*romR* Δ*frzE* Δ*aglQ*	[2]
SA12899	p_{Van}-*romR-mCherry* Δ*mglA* Δ*mglC* Δ*romR* Δ*frzE* Δ*aglQ*	This work
SA12895	*mglB*$^{K14A\ R115A\ K120A}$-*mCherry*	This work
SA12885	*mglB*$^{K14A\ R115A\ K120A}$-*mCherry* Δ*mglA*	This work
SA12896	*mglB*$^{K14A\ R115A\ K120A}$-*mCherry* Δ*mglC*	This work
SA12894	*mglA-mVenus mglB*$^{K14A\ R115A\ K120A}$	This work
SA12897	*mglC-mVenus mglB*$^{K14A\ R115A\ K120A}$	This work
SA12898	*romR-mCherry mglB*$^{K14A\ R115A\ K120A}$	This work
SA11080	*mglC*$^{F25A\ D26A\ I28A}$-*mVenus*	This work
SA12893	*mglA-mVenus* Δ*mglC* P_{pilA}-*mglC*$^{F25A\ D26A\ I28A}$	This work
SA12869	*mglB-mCherry* Δ*mglC* P_{pilA}-*mglC*$^{F25A\ D26A\ I28A}$	This work
SA12817	*romR-mCherry* Δ*mglC* P_{pilA}-*mglC*$^{F25A\ D26A\ I28A}$	This work
SA12892	*mglC*$^{K104A\ R106A\ R110A}$-*mVenus*	This work
SA12887	*mglC*$^{K104A\ R106A\ R110A}$-*mVenus* Δ*mglA*	This work
SA12890	*mglC*$^{K104A\ R106A\ R110A}$-*mVenus* Δ*mglB*	This work
SA12899	*mglC*$^{K104A\ R106A\ R110A}$-*mVenus* Δ*romR*	This work

SA12886	*mglC*$^{K104A\ R106A\ R110A}$*-mVenus* Δ*mglA* Δ*mglB*	This work
SA12889	*mglA-mVenus* Δ*mglC* P_{pilA}*-mglC*$^{K104A\ R106A\ R110A}$	This work
SA12891	*mglB-mCherry* Δ*mglC* P_{pilA}*-mglC*$^{K104A\ R106A\ R110A}$	This work
SA12888	*romR-mCherry* Δ*mglC* P_{pilA}*-mglC*$^{K104A\ R106A\ R110A}$	This work
SA10955	*romR*$^{1\text{-}368}$*-mCherry*	This work
SA10921	*romR*$^{1\text{-}368}$, *mglA-mVenus*	This work
SA10966	*romR*$^{1\text{-}368}$, *mglB-mCherry*	This work
SA11057	*romR*$^{1\text{-}368}$, *mglC-mVenus*	This work
SA13100	Δ*romR* P_{pilA}*-romR*^{C}*-mChery*	This work

Supplementary Table 2. Plasmids used in this work

Plasmids		Source or reference
pBJ114	In-frame deletion vector, Km^R	16
pSW105	Complementation from *attB* with P_{pilA}, Km^R	4
pAM5	P_{pilA} *mglC* Mx8 *attP*, Km^R	11
pLC66	pBJ114, construct for *mglC* replacement by *mglC-mVenus* at native site, Km^R	This work
pLC20	pBJ114, construct for *mglA* replacement by *mglA-mVenus* at native site, Km^R	15
pDK145	pBJ114, construct for *mglB* replacement by *mglB-mCherry* at native site, Km^R	14
pLC32	pBJ114, construct for *romR* replacement by *romR-mCherry* at native site, Km^R	15
pAM1	pBJ114 Δ*mglC,* construct for generation of in-frame deletion of *mglC*, Km^R	11
pSL16	pBJ114 Δ*mglA,* construct for generation of in-frame deletion of *mglA*, Km^R	12
pES2	pBJ114 Δ*mglB* construct for generation of in-frame deletion of *mglB,* Km^R	13
pSL37	pBJ114 Δ*romR*, construct for generation of in-frame deletion of *romR,* Km^R	14
pBJΔ*aglQ*	pBJ114 Δ*aglQ*; construct for generation of in-frame deletion of *aglQ,* Km^R	17
pLC155	pBJ114, construct for generation of in-frame deletion of Δ369-420 of *romR,* Km^R	This work
pLC154	pBJ114, construct for generation of in-frame deletion of Δ369-420 from *romR-mCherry,* Km^R	This work
pLC301	pBJ114, construct for *mglB* replacement by $mglB^{K14A\ R115A\ K120A}$ at native site, Km^R	This work
pLC280	pBJ114, construct for *mglB* replacement by $mglB^{K14A\ R115A\ K120A}$-*mCherry* at native site, Km^R	This work
pLC250	P_{pilA} $mglC^{F25A\ D26A\ I28A}$ Mx8 *attP*, Km^R	This work
pLC260	P_{pilA} $mglC^{K104\ R106\ R110}$ Mx8 *attP*, Km^R	This work
pLC295	pBJ114, construct for *mglC* replacement by $mglC^{F25A\ D26A\ I28A}$-*mVenus* at native site, Km^R	This work
pLC263	pBJ114, construct for *mglC* replacement by $mglC^{K104A\ R106A\ R110A}$-*mVenus* at native site, Km^R	This work
pLC1	P_{van} *romR-mCherry*, vanillate-dependent expression of *romR-mCherry* from *mxan18-19* locus, Tc^R	2
pASKIBA15+	Vector for overexpression of Strep-tagged proteins; Km^R	IBA Life-sciences GmbH
pLC242	Overexpression Strep-MglC, Amp^R	This work
pDK28	Overexpression MalE-RomR, Amp^R	14
pTM1	Overexpression MglA-His_6, Km^R	18
pTM2	Overexpression His_6-MglB, Km^R	18
pLC283	Overexpression His_6-$MglB^{K14A\ R115A\ K120A}$, Km^R	This work
pLC300	Overexpression MalE-$RomR^{1\text{-}368}$, Amp^R	This work
pLC310	Overexpression MalE-MglC, Amp^R	This work
pLC311	Overexpression MalE-$RomR^C$, Amp^R	This work
pLC312	P_{pilA} $romR^C$-*mCherry* Mx8 *attP*, Km^R	This work

Supplementary Table 3. Primers used in this work

**Primers (5'-3')**	
MglC_E	TTGGTGAAGCCCCCGTAACA
MglC_F	CTTGCCATTGTAGAAGAGGA
MglC_G	AGC GTG ATG GGC TTT GAC GGC ATC
MglC_H_2	TAC CAT CCG CGT GAA GGG CGA GCA CCA
MglC_FW	ATCG AAG CTT AGG CCA CGT ACC CCG TCA
MglC_Link_RV	CTC GCC CTT GCT CAC CAT GAG CTC GGC GCG CAC CTT
MglCDS800_FW	ATCG TCT AGA TCG GAT GCC CGG CCG
MglCDS800_RV	ATCG GAA TTC CGC CTG GGC CCG GGT
RomR_FW_HindIII	ATCG AAG CTT CGC CGG GGG CCC GTC
RomR_Cterm_B	CCA GGC GCC TCA AGG CGC ACG GGC GCT CGC CGG
RomR_Cterm_C	GCG CCT TGA GGC GCC TGG CGC CGT AAC CTC
RomR_dsRV_EcoRI	ATCG GAA TTC ATC AGG TCC TGG TAG CGC TCG TC
R_Cterm_B_linkerlessgc	TGA TCC ACC GCC TCC AGG CGC ACG GGC GCT CGC CGG
Venus_FW_LessGC	GGA GGC GGT GGA TCA ATG GTG AGC AAG GGC GAG
GFP RV EcoRI	ATCG GAA TTC TTA CTT GTA CAG CTC GTC CAT GCC G
MglC_FA/DA/IA_UP	TGC AGC GTG ATG GGC GCT GCC GGC GCC TCC
MglC_FA/DA/IA_DS	GAA GGT GTC GAC GGA GGC GCC GGC AGC GCC
MglC_KA/RA/RA_UP	TTC GGC GCG GGC GCC TAC GTG CTG GCC GTC ACT
MglC_KA/RA/RA_DS	AGT GAC GGC CAG CAC GTA GGC GCC CGC GCC GAA
MglAB_natprom_fw	ATCG AAG CTT CGTCGTTCGGCGTGAAGCCCT
mglb_k14_rv	AAA CGG CGT TGA TCG CGG TGA ACT CCT
mglb_k14_fw	AGG AGT TCA CCG CGA TCA ACG CCG TTT
mglb_r105k120_rv	GTC GCT GGC CGC CTT GAT GCG AAG GGC GAC GAG GC
mglb_r105k120_fw	GGC CTC GTC GCC CTT CGC ATC AAG GCG GCC AGC GA
MglA_RV_EcoRI	ATC GGA ATT CTC AAC CAC CCT TCT TGA
MglC_fw_xbaI	ATC TCT AGA ATG TCC TTC CGC ACG CAC CTC
MglC_rv_HindIII	ATC AAG CTT CTA GAG CTC GGC GCG CAC CTT
MglC_fw_EcoRI	ATC GAA TTC ATG TCC TTC CGC ACG CAC CTC GAG
romr_fw_EcoRI	ATCG GAA TTC ATG CCC AAG AAT CTG CTG GTC GC
romr_368_rv_hindIII	ATC AAG CTT AGG CGC ACG GGC GCT CGC CGG
romr_369_fw_EcoRI	ATC GAA TTC ATG GCC GCG GAT GGG GGC GAG GCC
romr_rv_hindIII	ATC AAG CTT GTG CTG GGT CTC TCG GTC CTT GAT
romr_369_fw_XbaI	ATC TCT AGA ATG GCC GCG GAT GGG GGC GAG GCC
mCherry_RV_HindIII	ATCG AAG CTT TTA CTT GTA CAG CTC GTC CAT GCC

Supplementary Table 4. Quantification of polar fluorescence signals

Strain	Mean fraction of fluorescence at pole 1	Mean fraction of fluorescence at pole 2	Fraction of cells w/two clusters	Fraction of cells w/one cluster
mglC-mVenus	0.35	0.14	0.99	0.01
mglC-mVenus ΔmglA	0.57	0.05	0.79	0.20
mglC-mVenus ΔmglB	0.13	0.07	1.00	0.00
mglC-mVenus ΔromR	0.01	0.00	0.19	0.68
mglA-mVenus	0.01	0.00	0.55	0.37
mglA-mVenus ΔmglC	0.00	0.00	0.09	0.38
mglB-mCherry	0.07	0.03	0.90	0.10
mglB-mCherry ΔmglC	0.02	0.00	0.36	0.55
romR-mCherry	0.20	0.07	0.98	0.02
romR-mCherry ΔmglC	0.13	0.05	0.92	0.07
mglC-mVenus ΔmglA ΔmglB	0.20	0.12	0.98	0.02
mglB-mCherry ΔmglA	0.22	0.05	0.77	0.22
mglB-mCherry ΔmglA ΔmglC	0.00	0.00	0.00	0.06
romR-mCHerry ΔmglA	0.49	0.08	0.91	0.09
romR-mCherry ΔmglB	0.09	0.04	0.96	0.04
romR-mCherry ΔmglB ΔmglC	0.08	0.04	0.92	0.08
romR-mCherry ΔmglA ΔmglB	0.14	0.06	0.89	0.11
romR-mCherry ΔmglA ΔmglC	0.11	0.05	0.92	0.08
romR-mCherry ΔmglA ΔmglB ΔmglC	0.08	0.04	0.92	0.08
mglB-mCherry ΔmglC ΔromR	0.00	0.00	0.00	0.07
mglC-mVenus ΔmglA ΔromR	0.04	0.00	0.25	0.69
mglC-mVenus ΔmglB ΔromR	0.01	0.00	0.18	0.67
mglC-mVenus ΔmglA ΔmglB ΔromR	0.00	0.00	0.00	0.01
mglC^{FDI}-mVenus	0.21	0.09	0.97	0.03
mglA-mVenus ΔmglC psw105-mglC^{FDI}	0.00	0.00	0.08	0.47
mglB-mCherry ΔmglC psw105-mglC^{FDI}	0.02	0.01	0.69	0.28
romR-Cherry ΔmglC psw105-mglC^{FDI}	0.12	0.07	0.97	0.03
mglC^{FDI}-mVenus ΔmglA	0.19	0.12	1.00	0.00
mglC^{FDI}-mVenus ΔmglB	0.15	0.07	0.99	0.01
mglC^{FDI}-mVenus ΔromR	0.00	0.00	0.00	0.01
romR-Cherry ΔmglC psw105-mglC^{KRR}	0.13	0.04	0.93	0.07
mglA-mVenus ΔmglC psw105-mglC^{KRR}	0.00	0.00	0.00	0.10
mglB-mCherry ΔmglC psw105-mglC^{KRR}	0.04	0.01	0.76	0.24
mglC^{KRR} mVenus	0.01	0.00	0.04	0.69
mglC^{KRR} mVenus ΔromR	0.00	0.00	0.00	0.11
mglC^{KRR} mVenus ΔmglA	0.02	0.00	0.36	0.57
mglC^{KRR} mVenus ΔmglB	0.00	0.00	0.00	0.02
mglB^{KRK}-mCherry	0.00	0.00	0.00	0.09
mglB^{KRK}-mCherry ΔmglA	0.00	0.00	0.00	0.10
mglB^{KRK}-mCherry ΔmglC	0.00	0.00	0.06	0.32
romR$^{1-368}$-Cherry	0.00	0.00	0.00	0.23
mglA-mVenus romR$^{1-368}$	0.01	0.00	0.06	0.54
mglB-mCherry romR$^{1-368}$	0.00	0.00	0.02	0.19
mglC-mVenus romR$^{1-368}$	0.01	0.00	0.19	0.49

Δ*romR* P_{pilA}-*romR*^{C}-*mCherry*	0.01	0.00	0.13	0.71

Supplementary References

1. Shi, W. & Zusman, D.R. The two motility systems of *Myxococcus xanthus* show different selective advantages on various surfaces. *Proc. Natl. Acad. Sci. USA* **90**, 3378-3382 (1993).

2. Carreira, L.A.M., Tostevin, F., Gerland, U. & Søgaard-Andersen, L. Protein-protein interaction network controlling establishment and maintenance of switchable cell polarity. *PLOS Genetics* **16**, e1008877 (2020).

3. Bischof, L.F., Friedrich, C., Harms, A., Søgaard-Andersen, L. & van der Does, C. The type IV pilus assembly ATPase PilB of *Myxococcus xanthus* interacts with the inner membrane platform protein PilC and the nucleotide-binding protein PilM. *J. Biol. Chem.* **291**, 6946-6957 (2016).

4. Jakovljevic, V., Leonardy, S., Hoppert, M. & Søgaard-Andersen, L. PilB and PilT are ATPases acting antagonistically in type IV pili function in *Myxococcus xanthus*. *J. Bacteriol.* **190**, 2411-2421 (2008).

5. Galicia, C. *et al.* MglA functions as a three-state GTPase to control movement reversals of *Myxococcus xanthus*. *Nat. Commun.* **10**, 5300 (2019).

6. Kapoor, S., Kodesia, A., Kalidas, N., Ashish & Thakur, K.G. Structural characterization of *Myxococcus xanthus* MglC, a component of the polarity control system, and its interactions with its paralog MglB. *J. Biol. Chem.* **296**, 100308 (2021).

7. Baranwal, J. *et al.* Allosteric regulation of a prokaryotic small Ras-like GTPase contributes to cell polarity oscillations in bacterial motility. *PLOS Biol.* **17**, e3000459 (2019).

8. Kaiser, D. Social gliding is correlated with the presence of pili in *Myxococcus xanthus*. *Proc. Natl. Acad. Sci. USA* **76**, 5952-5956 (1979).

9. Wu, S.S. & Kaiser, D. Markerless deletions of *pil* genes in *Myxococcus xanthus* generated by counterselection with the *Bacillus subtilis sacB* gene. *J. Bacteriol.* **178**, 5817-5821 (1996).

10. Jakobczak, B., Keilberg, D., Wuichet, K. & Søgaard-Andersen, L. Contact- and protein transfer-dependent stimulation of assembly of the gliding motility machinery in *Myxococcus xanthus*. *PLOS Genet* **11**, e1005341 (2015).

11. McLoon, A.L. *et al.* MglC, a paralog of *Myxococcus xanthus* GTPase-Activating Protein MglB, plays a divergent role in motility regulation. *J. Bacteriol.* **198**, 510-520 (2016).

12. Miertzschke, M. *et al.* Structural analysis of the Ras-like G protein MglA and its cognate GAP MglB and implications for bacterial polarity. *EMBO J.* **30**, 4185-4197 (2011).

13. Leonardy, S. *et al.* Regulation of dynamic polarity switching in bacteria by a Ras-like G-protein and its cognate GAP. *EMBO J.* **29**, 2276-2289 (2010).

14. Keilberg, D., Wuichet, K., Drescher, F. & Søgaard-Andersen, L. A response regulator interfaces between the Frz chemosensory system and the MglA/MglB GTPase/GAP module to regulate polarity in *Myxococcus xanthus*. *PLOS Genet.* **8**, e1002951 (2012).

15. Szadkowski, D. *et al.* Spatial control of the GTPase MglA by localized RomR/RomX GEF and MglB GAP activities enables *Myxococcus xanthus* motility. *Nat. Microbiol.* **4**, 1344-1355 (2019).

16. Julien, B., Kaiser, A.D. & Garza, A. Spatial control of cell differentiation in *Myxococcus xanthus*. *Proc. Natl. Acad. Sci. USA* **97**, 9098-9103 (2000).

17. Sun, M., Wartel, M., Cascales, E., Shaevitz, J.W. & Mignot, T. Motor-driven intracellular transport powers bacterial gliding motility. *Proc. Natl. Acad Sci. USA* **108**, 7559-7564 (2011).

18. Zhang, Y., Franco, M., Ducret, A. & Mignot, T. A bacterial Ras-like small GTP-binding protein and its cognate GAP establish a dynamic spatial polarity axis to control directed motility. *PLOS Biol* **8**, e1000430 (2010).
