# Supplementary material for: Molecular basis and design principles of switchable front-rear polarity and directional migration in Myxococcus xanthus
Source: Nat Commun. 2023 Jul 8;14:4056. doi: 10.1038/s41467-023-39773-y (PMC10329633; doi:10.1038/s41467-023-39773-y)
Supplement: Supplementary file 3 — Reporting Summary [file 41467_2023_39773_MOESM3_ESM.pdf]

nature portfolio

Corresponding author(s): Lotte Søgaard-Andersen

Last updated by author(s): 2023/06/22

Reporting Summary

Nature Portfolio wishes to improve the reproducibility of the work that we publish. This form provides structure for consistency and transparency in reporting. For further information on Nature Portfolio policies, see our Editorial Policies and the Editorial Policy Checklist.

Statistics

For all statistical analyses, confirm that the following items are present in the figure legend, table legend, main text, or Methods section.

n/a	Confirmed	
☐	☒	The exact sample size (*n*) for each experimental group/condition, given as a discrete number and unit of measurement
☐	☒	A statement on whether measurements were taken from distinct samples or whether the same sample was measured repeatedly
☐	☒	The statistical test(s) used AND whether they are one- or two-sided<br>*Only common tests should be described solely by name; describe more complex techniques in the Methods section.*
☒	☐	A description of all covariates tested
☒	☐	A description of any assumptions or corrections, such as tests of normality and adjustment for multiple comparisons
☐	☒	A full description of the statistical parameters including central tendency (e.g. means) or other basic estimates (e.g. regression coefficient) AND variation (e.g. standard deviation) or associated estimates of uncertainty (e.g. confidence intervals)
☐	☒	For null hypothesis testing, the test statistic (e.g. *F*, *t*, *r*) with confidence intervals, effect sizes, degrees of freedom and *P* value noted<br>*Give P values as exact values whenever suitable.*
☒	☐	For Bayesian analysis, information on the choice of priors and Markov chain Monte Carlo settings
☒	☐	For hierarchical and complex designs, identification of the appropriate level for tests and full reporting of outcomes
☒	☐	Estimates of effect sizes (e.g. Cohen's *d*, Pearson's *r*), indicating how they were calculated

Our web collection on statistics for biologists contains articles on many of the points above.

Software and code

Policy information about availability of computer code

Data collection	Bacterial microscopy was performed with LAS-X (3.7.6.25997) (Leica) and NIS Elements AR 2.30 software (Nikon). Infinite M200 Pro plate-reader data acquisition was done using Tecan i-control 3.7.3.0, Mass photometry data were collected using AcquireMP (Refeyn Ltd. v2.3), AlphaFold structures were generated using the ColabFold pipeline (Mirdita et al.,2022).
Data analysis	The following software was used for data analysis: ImageJ 1.52b (Schneider et al., 2012), Fiji (Schindelin et al., 2012), MetaMorph 7.5 (molecular Devices), Oufti (Paintdakhi et al., 2016), MATLAB R2020a (The MathWorks), Pymol (The PyMOL Molecular Graphics System, Version 1.2r3pre, Schrödinger, LLC), DiscoverMP software (Refeyn Ltd, v. 2022 R1).

For manuscripts utilizing custom algorithms or software that are central to the research but not yet described in published literature, software must be made available to editors and reviewers. We strongly encourage code deposition in a community repository (e.g. GitHub). See the Nature Portfolio guidelines for submitting code & software for further information.

Data

Policy information about availability of data

All manuscripts must include a data availability statement. This statement should provide the following information, where applicable:

- Accession codes, unique identifiers, or web links for publicly available datasets
- A description of any restrictions on data availability
- For clinical datasets or third party data, please ensure that the statement adheres to our policy

The data that supports the findings of this study are available from the corresponding author upon request.

Human research participants

Policy information about studies involving human research participants and Sex and Gender in Research.

Reporting on sex and gender	N/A
Population characteristics	N/A
Recruitment	N/A
Ethics oversight	N/A

Note that full information on the approval of the study protocol must also be provided in the manuscript.

Field-specific reporting

Please select the one below that is the best fit for your research. If you are not sure, read the appropriate sections before making your selection.

☒ Life sciences ☐ Behavioural & social sciences ☐ Ecological, evolutionary & environmental sciences

For a reference copy of the document with all sections, see nature.com/documents/nr-reporting-summary-flat.pdf

Life sciences study design

All studies must disclose on these points even when the disclosure is negative.

Sample size	No statistical methods were used to predetermine the sample size. The sample size was determined based on our expertise in bacterial cell biology. Generally, three biological replicates with a high number of cells (n) were used for the image analysis of fluorescence pictures. For single cell motility, three biological replicates with a minimum of 20 cells were analyzed. Similarly, we decided that based on our own experience, for in vitro experiments, several independent protein purifications served as biological replicates.
Data exclusions	No data were excluded.
Replication	All experiments were successfully replicated. The nature and number of replicates is indicated in the corresponding figure legend. Informations provided in the Methods section are sufficient to reproduce all the experiments. All single cell data presented is representative of the population.
Randomization	The experiments were not randomized, since there was no allocation into subgroups.
Blinding	No blinding was performed because the acquisition and analysis required human intervention.

Reporting for specific materials, systems and methods

We require information from authors about some types of materials, experimental systems and methods used in many studies. Here, indicate whether each material, system or method listed is relevant to your study. If you are not sure if a list item applies to your research, read the appropriate section before selecting a response.

Materials & experimental systems

n/a	Involved in the study
☐	☒ Antibodies
☒	☐ Eukaryotic cell lines
☒	☐ Palaeontology and archaeology
☒	☐ Animals and other organisms
☒	☐ Clinical data
☒	☐ Dual use research of concern

Methods

n/a	Involved in the study
☒	☐ ChIP-seq
☒	☐ Flow cytometry
☒	☐ MRI-based neuroimaging

Antibodies

Antibodies used

All antibodies used in this study are described in the Methods section. A rabbit polyclonal antibody against MglC was generated as described in Methods and used in dilution 1:5000. Other antibodies used in this study include rabbit α-MglA (Leonardy et al., 2010) (dilution 1:5000), rabbit α-MglB (Leonardy et al., 2010) (dilution 1:5000), rabbit α-RomR (Leonardy et al., 2007) (dilution 1:5000), rabbit α-PilC (Bulyha et al., 2009) (dilution 1:5000), rabbit α-PilO (Friedrich et al., 2014) (1:2000 dilution), rabbit α-PilT (Jakovljevic et al. 2008) (dilution 1:2000), rabbit α-mCherry (Biovision, cat.nr. 5993-100) (dilution 1:15000), mouse α-EF-Tu (HycultBiotech, cat.nr. HM6010) (dilution 1:5000) and mouse α-GFP (Sigma, cat.nr. G6539) (dilution 1:2000), goat α-rabbit immunoglobulin G peroxidase conjugate (Sigma, cat.nr. A9044) (dilution 1:10000), sheep α-mouse immunoglobulin G, horseradish peroxidase lined whole antibody (GE Healthcare, cat.nr. NA931-1ML) (dilution 1:2000).

Validation

Data provided in the manuscript confirmed specificity of used antibodies, α-MglC specifically reacts with MglC, MglC-mVenus (Supplementary Figure 1b,e); α-MglA with MglA (Supplementary Figure 1d); α-MglB with MglB (Supplementary Figure 1d); α-RomR with RomR (Supplementary Figure 1d); α-GFP with MglC-mVenus (Supplementary Figure 1b); α-PilC with PilC (Supplementary Figure 1b), α-PilO with PilO (Supplementary Figure 5f), α-PilT with PilT-His6 (Supplementary Figure 5b), α-mCherry with mCherry (Supplementary Figure 5f) and α-EF-Tu with EF-Tu (Supplementary Figure 5b).

March 2021
